# Cultivar Differences in the Biochemical and Physiological Responses of Common Beans to Aluminum Stress

**DOI:** 10.3390/plants10102097

**Published:** 2021-10-03

**Authors:** Brigitta Tóth, Makoena Joyce Moloi, Lóránt Szőke, Mátyás Danter, Michael A. Grusak

**Affiliations:** 1 Institute of Food Science, University of Debrecen, 138 Böszörményi St., 4032 Debrecen, Hungary; szoke.lorant@agr.unideb.hu; 2 Department of Plant Sciences, University of the Free State-Main Campus, P.O. Box 339, Bloemfontein 9300, South Africa; MoloiMJ@ufs.ac.za; 3 Modern Application Platform Business Unit, VMware, Inc., 3401 Hillview Ave, Palo Alto, CA 94304, USA; mdanter@gmail.com; 4 USDA-ARS Edward T. Schafer Agricultural Research Center, 1616 Albrecht Boulevard N., Fargo, ND 58102-2765, USA; mike.grusak@usda.gov

**Keywords:** aluminum toxicity, antioxidant enzyme activity, bean, lipid peroxidation, low pH, reactive oxygen species

## Abstract

Soil conditions leading to high levels of available aluminum are detrimental to plant growth, but data are limited on genotypic differences in tolerance to aluminum stress in some crops. The aim of this study was to examine the morphological, biochemical, and physiological changes in roots and shoots of 25 common bean (*Phaseolus vulgaris* L.) cultivars (Pinto market class) under aluminum (Al) treatment. Additionally, this study aimed to assess the range of responses amongst the common bean cultivars relative to their Al toxicity tolerance and sensitivity. Plants were grown hydroponically using a simplified nutrient solution with or without 20 µM AlCl_3_. Reactive oxygen species (ROS), activities of the antioxidant enzymes superoxide dismutase (SOD) and guaiacol peroxidase (POD), and malondialdehyde (MDA) concentration, an indicator of lipid peroxidation, were measured to establish the effects of Al treatment on the plants. In addition, growth parameters such as shoot and root dry weight, root-to-shoot ratio, root elongation, and root volume changes were also investigated. The cultivar effect was significant for all the measured parameters, except for shoot dry weight. Inhibition of the root and shoot dry weight for selected common bean cultivars shows that the response of common bean to Al stress is genotype-specific. Additionally, Al-induced root elongation inhibition and root volume changes varied among the cultivars. Most cultivars had significantly higher SOD activity (20 of 25 cultivars) and POD activity (12 cultivars) under AlCl_3_ treatment compared to the controls. A positive significant correlation was observed between MDA and ROS, showing that Al stress induced the accumulation of ROS along with an increase in lipid peroxidation. According to the results of this study, Arapaho and AC Island cultivars could potentially be used in the future production of common beans under Al stress. Therefore, these two cultivars could also be included in Al tolerance breeding programs.

## 1. Introduction

Climate change is an important topic that dates back to the early 1950s. Although there were arguments regarding its existence, a consensus was reached about its impact on nature, environment, plants, animals, and humans. Climate change has direct and indirect impacts on crop production and food security. Water imbalance (drought and flooding), elevated CO_2_ concentration in the atmosphere, increasing average annual global temperature, extreme weather conditions, and decline in soil properties are the main features of climate change related to agriculture. Agricultural lands are among the first affected by climate change [1]. Climate change affects soil pH and the availability of different macro- and micro-elements [2]. Anthropogenic features such as increasing greenhouse gas emissions, elevated atmospheric carbon dioxide concentration, nitrogen overfertilization of soils, and acid rain contribute to soil acidity [3] and, consequently, promote aluminum (Al) toxicity. Climate change is also linked to extreme weather conditions such as acidic rainfall. The pH of rain has been changing in many regions in the last decades. The pH of rain is normally slightly acidic and ranges between 5.0 and 6.0 [4]. If the rainfall contains sulfur dioxide or nitrogen oxides—as a consequence of anthropogenic environmental pollution—the pH drops to 4.0 or lower in extreme cases [5]. The effects of acid rain on soil acidification depend on soil properties, as well as soil buffer capacity. This soil characteristic is related to the composition of the soil and the soil coverage of the surface. Especially in the northeast part of the United States of America, where the soil is thin and buffer capacity is low, acid rain generates soil acidification and accelerates the dissolution of aluminum salts, which induces aluminum stress [6]. Aluminum mineral salts, i.e., aluminum oxides and aluminosilicates, in soils are not toxic to the plants on their own. Under acidic conditions, Al minerals form toxic Al-hydroxide. The most toxic form of Al is Al^3+^ which has the greatest impact on plant growth [7]. In addition, tropical and subtropical soils—a significant proportion of global soils—are highly sensitive to soil acidification because of their low buffer capacity [8]. The average Al concentration in soil varies between 0.01 and 0.3 ppm [9]. Aluminum becomes toxic when the soil pH drops below 5.5 and Al concentration increases in the soil solution. Soil solution at neutral pH contains 400 µg·L^−1^ Al, while this value can be 5700 µg·L^−1^ in 4.4 pH soil [10]. If the Al concentration in soil solution is higher than 1 mg·L^−1^, aluminum toxicity and reduced yield can occur. A soil aluminum concentration of 2–5 ppm is toxic to the roots of sensitive plant species, and a concentration above 5 ppm is toxic to tolerant species [11]. The Al concentrations measured in plant tissues are different from the soil Al concentrations. The average Al concentration in plants is in the 10 s and 100 s of mg·kg^−1^, while this value is in the 1000 s of mg·kg^−1^ in Al accumulator species. Plants can accumulate or exclude Al from their metabolism. Accumulator species mainly occur in the tropical and subtropical regions, accumulating a minimum of 1 g Al·kg^−1^ in the dry leaf tissue [12]. For instance, buckwheat can accumulate more than 15 g Al·kg^−1^ in leaves growing on acidic soil [13]. The plants which exclude Al from their metabolisms secrete metabolites, i.e., organic acids, which form nontoxic chelates with Al [13]. To protect our crops from the toxic effects of Al, liming is the most widely used agricultural practice [14]. Numerous studies indicate that the toxicity of Al is one of the principal abiotic stressors, especially under acidic growing conditions, which impacts crop production and yield at physiological and morphological levels [15,16,17,18,19]. The most sensitive plant organ is the root, and the first symptom of Al toxicity is reduced root growth [20]. The degree of the effects of Al on plant growth and development depends on the concentration of Al, species, genotype, cultivar, and duration of Al exposure [21,22]. Furthermore, Al can bind with the cell wall, whereby the cell wall becomes rigid [23]. Aluminum can change the lipid peroxidation in the plasma membrane [24] and the homeostasis of calcium ions [25], inhibit the uptake of nutrients and water [7], and decrease the chlorophyll content [26], and photosynthetic rate [27].

Similar to many abiotic stressors, Al toxicity stimulates the generation of reactive oxygen species (ROS) in plant cells and, consequently, oxidative stress [28,29,30,31]. The ROS are highly reactive, and their overproduction is toxic to biomolecules. To protect the biomolecules under Al toxicity, a highly effective antioxidant enzyme system is required [32,33]. One of the key constituents of this defense system is represented by metalloenzymes; superoxide dismutase (SOD) enzymes belong to this group. Elevated activities of SOD were measured in Al-tolerant rice [32], Barbados nut [34], wheat [35], tomato [36], and soybeans [37]. Another antioxidant enzyme, ascorbate peroxidase (APX), is one of the most effective controllers of ROS because it plays a significant role in hydrogen peroxide detoxification [38]. Rajput et al. [39] communicated that the activity of APX increases with activities of other enzymatic antioxidants such as SOD or glutathione reductase (GR), indicating their interdependence. Although reports have indicated increased APX activity in crops under different abiotic stress conditions such as drought stress in maize [40], wheat [41], and peas [42], there is conflicting evidence under Al stress. For example, Al stress induced an insignificant increase in the APX activity of tolerant rice with higher activity in sensitive plants [32]. Du et al. [37] reported a higher APX activity in ZmAT6 transgenic maize, while no change was observed in the OE-ZmAT6 line after aluminum treatment. On the contrary, Al stress induced a significant increase in the APX activity of sensitive compared to tolerant wheat in their root tips [43]. Noteworthy, however, is that the activity of APX also depends on the duration and intensity of stress [44]. Guaiacol peroxidase (POD) is another member of plants’ detoxifying systems, as it plays a key role in the removal of hydrogen peroxide generated during stress conditions in plants. Peroxidase is not always a biochemical marker of Al tolerance because its activity was found to be notably higher in Al-sensitive than in Al-resistant maize [45]. Additionally, increased POD activity has been documented in several research studies under different environmental conditions [32,46,47,48,49,50].

Common beans (*Phaseolus vulgaris* L.) are important protein sources in the vegetarian diet and in developing countries. Beans have a high carbohydrate and low fat content, are rich in fiber, and have a low glycemic index [51]. Approximately 8.96 million ha of land is used for growing beans in Latin America, and over 4 million ha is used on the African continent [52,53]. Aluminum toxicity is one of the principal restricting components of common bean production in tropical regions [54], leading to a significant yield reduction in these territories [55]. In the context of bean production, Al soil toxicity mostly affects small-scale farms [55,56]. Since the common bean is sensitive to Al stress [57], several solutions can be implemented to avoid yield loss under Al toxicity. These solutions include cultivation in acidic soil and the use of aluminum-resistant or tolerant lines and genotypes [58,59].

It is difficult to categorize plant species according to their Al tolerance. Pineapple and tea are known as Al-tolerant plants, while most plant species are Al-sensitive, but many wild plant species have adapted to acidic soil and high Al concentration. The degree of sensitivity depends on the origin of plants, the species, and the soil properties [37]. Two geographical origins of the gene pools of common bean are Mesoamerica and the Andes. Accordingly, the common bean cultivars are separated into two groups called Mesoamerican and Andean races beans [60]. Common beans were cultivated on acidic soils in Spain from the 17th century. According to research data, a connection was found between these Spanish beans and the Andean race bean cultivars [61]. On the one hand, these data suggest that common beans adapted to acidic soil conditions, which exist in Central and South America [62]. On the other hand, Lunze et al. [63] stated that the common bean is sensitive to strongly acidic soil conditions; its extremes are soil with pH under 5.0 and above 8.0. In addition, the common bean is an Al-sensitive plant according to Horneck et al.’s [64] data.

The responses of common beans to Al toxicity have been researched widely. The first visible Al toxicity deformation involves root growth retardation [65]. Llugany et al. [66] observed significant root growth inhibition in maize 30–90 min after Al treatment. Similarly, short-term exposure to Al caused root elongation inhibition in common beans [67]. Massot et al. [68] stated that callose synthesis negatively correlated with root elongation rate after 24 h of Al exposure. Additionally, the root elongation rate is the most noticeable parameter used to observe the effect of Al on bean cultivars. The extent of root damage caused by Al is based on the stage of plant development and growth, the concentration of Al, and the degree of Al tolerance of plants [69] at the time of its exposure to Al.

This experiment used 25 common bean genotypes of the Pinto market class. The goal of this research was to examine the physiological, biochemical, and morphological changes in roots, the concentration of reactive oxygen species (ROS), the activity of antioxidant enzymes such as superoxide dismutase (SOD) and guaiacol peroxidase (POD), and the rate of lipid peroxidation in seedlings growing under AlCl_3_ toxicity. The additional aim was to assess the range of responses amongst the common bean cultivars relative to their Al toxicity tolerance and sensitivity.

## 2. Results

The root dry weight was significantly lower in all Al-treated cultivars, except AC Island and Arapaho. The root dry weight varied between 57 and 104 mg·plant^−1^ in nontreated plants, while this range was 25–78 mg·plant^−1^ in plants treated with AlCl_3_. More than a 50% decline in root dry weight was measured in Aztec (56%), Burke (51%), Croissant 56%), Kimberly (57%), Quincy (61%), and TARS-09 (55%). Ouray had the lowest reduction (23%). The effect of Al treatment was more noticeable on the root than the shoot dry weight. The shoot dry weight of Al-treated Aztec was 15% lower than that of the control. In contrast, Al treatment significantly increased shoot dry weight by 11% in Montrose. The root-to-shoot ratio was higher for the controls (ranged between 0.19 and 0.43) than that of the Al-treated cultivars (0.09–0.31). Although the root-to-shoot ratio was significantly reduced for most cultivars under Al treatment, AC Island and Arapaho were not affected (Table 1).

Aluminum treatment had less of an effect on the percentage change in the root volume of Arapaho for the entire experimental period. The changes ranged between +10% (Arapaho) and −81% (Burke) 24 h after Al treatment. Forty-eight hours after Al treatment, Arapaho exhibited the lowest change in root percentage (−39%) and Kodiak exhibited the highest (−90%). Similarly, after 72 h of Al treatment, Arapaho had the lowest percentage change (−52%) while Kodiak had the highest (−92%) (Table 2).

The Al-induced primary root inhibition varied between 15.25% (Burke) and 72.39% (Buckskin) 4 h after Al treatment. Root growth inhibition was higher 8 h after Al treatment, increasing from 36.67% to 86.00%, compared to the values 4 h after Al treatment. The lowest inhibition was measured in Arapaho 24 h after the Al treatment, while the highest was recorded in Poncho (91.35%). The root length inhibition was between 7.88% and 95.00% 48 h, and between 35.43% and 96.15% 72 h after Al treatment (Table 3).

**Figure 1:**
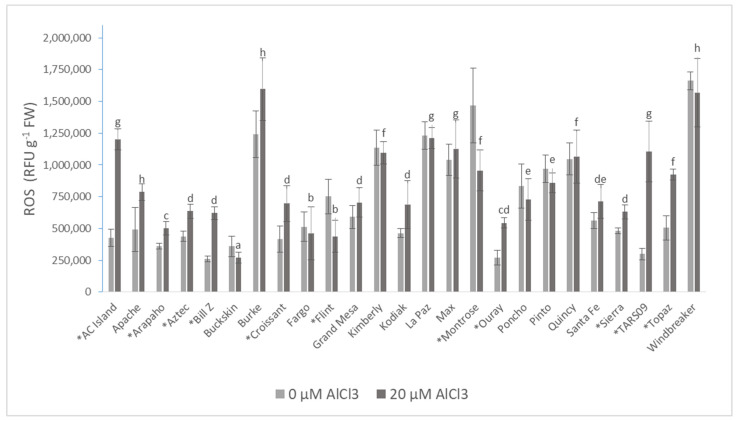
The total reactive oxygen species (ROS) of 25 common bean cultivars treated for 72 h with AlCl_3_. Values are the means of five biological and technical repetitions ± S.D. * Significant difference between treatments based on Shapiro–Wilk test (*p* ≤ 0.05). Lowercase letters denote a significant difference among the AlCl_3_-treated cultivars. FW: fresh weight, RFU: relative fluorescence unit.

The concentration of reactive oxygen species (ROS) varied widely in the control bean cultivars: 261,711 RFU·g^−1^ FW (Bill Z control) and 1,661,220 RFU·g^−1^ FW (Windbreaker control). The concentration of total ROS was 1.5 times (i.e., significantly) lower in Montrose grown in nutrient solution containing 20 µM AlCl_3_, compared to the control treatment. The effect of Al treatment was by far the highest in TARS09 (72.99% increase) and AC Island (64.49% increase) compared to the controls. In contrast, Flint and Montrose had remarkably lower ROS under Al treatment (41.93% and 34.84%, respectively) (Figure 1).

**Figure 2:**
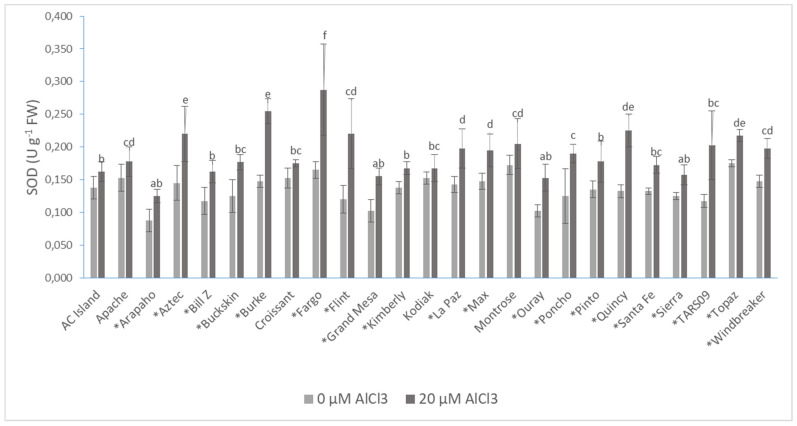
The superoxide (SOD) activity of 25 common bean cultivars 72 h after AlCl_3_ treatment. Values are the averages of five biological and technical repetitions ± SD. * Significant difference between treatments based on Shapiro–Wilk test (*p* ≤ 0.05). Lowercase letters denote a significant difference among the AlCl_3_-treated cultivars. FW: fresh weight.

Higher superoxide dismutase (SOD) activities were measured in the Al-treated roots of common beans compared to the controls. The activity of SOD was significantly higher in Arapaho, Aztec, Bill Z, Burke, Fargo, Flint, Grand Mesa, Kimberly, La Paz, Max, Ouray, Poncho, Pinto, Quincy, Santa Fe, Sierra, TARS09, and Topaz cultivars. Flint had the highest increase in SOD activity (45.5%) under Al treatment (Figure 2).

**Figure 3:**
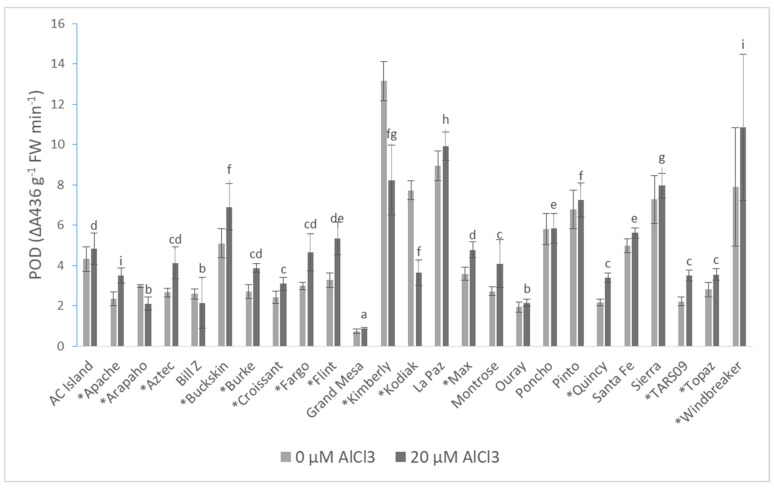
The peroxidase (POD) activity of 25 common bean cultivars 72 h after AlCl_3_ treatment. Values are the averages of five biological and technical repetitions ± SD. * Significant difference between treatments based on Shapiro–Wilk test (*p* ≤ 0.05). Lowercase letters denote a significant difference among the AlCl_3_-treated cultivars. FW: fresh weight.

Peroxidase (POD) activity varied among the cultivars under Al treatment. The lowest activity was measured in Grand Mesa (0.878 g^−1^ FW·min^−1^), while the highest was measured in Windbreaker (10.848 g^−1^ FW·min^−1^). Kodiak had the greatest reduction in POD activity (52.76%) under Al treatment, followed by Kimberly (37.33%) and Arapaho (29.62%). In contrast, POD activity was higher by more than 30% in Al-treated cultivars of Apache, Aztec, Burke, Fargo, Flint, Quincy, and TARS-09 compared to the control plants (Figure 3).

**Figure 4:**
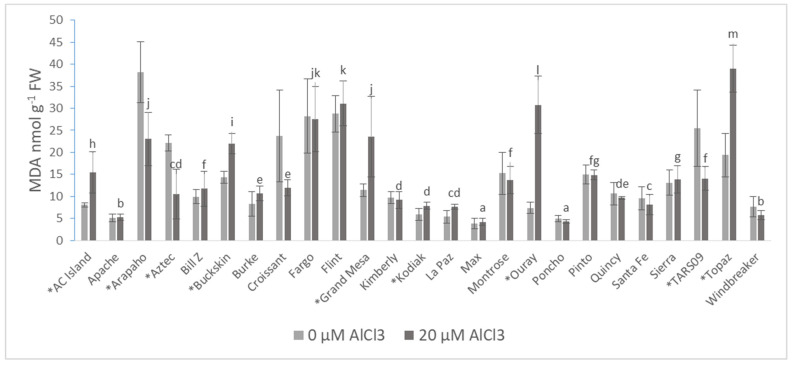
The malondialdehyde (MDA) concentrations of 25 common bean cultivars 72 h after AlCl_3_ treatment. Values are the averages of five biological and technical repetitions ± SD. * Significant difference between treatments based on Shapiro–Wilk test (*p* ≤ 0.05). Lowercase letters denote a significant difference among the AlCl_3_-treated cultivars. FW: fresh weight. MDA: malondialdehyde.

To evaluate the rate of lipid peroxidation, the concentration of malondialdehyde (MDA) was measured in the roots of common beans. Max had the lowest (4.197 nmol·g^−1^ FW) and Topaz had the highest (39.02 nmol·g^−1^ FW) MDA content under Al treatment. Compared to the control, the highest increases were measured in the Ouray (76.05%), Grand Mesa (51.54%), and Topaz (50.42%) cultivars. In contrast, Al treatment led to significant reductions in the MDA contents of Arapaho (39.78%), Aztec (52.65%), and TARS-09 (44.66%) (Figure 4).

To demonstrate the impact of Al on the measured parameters more noticeably, Table 4 contains the mean values of the measured parameters of 25 common bean cultivars. The average root dry weight was significantly lower (37.5%), while the average shoot dry weight was not affected by the Al treatment. Furthermore, the average value of root-to-shoot ratio also was significantly lower (by 42%) when Al treatment was examined. The average root volume and average root length significantly declined, with this rate of reduction decreasing as time went on. The changes in root volume were significant 24 (51.70%), 48 (72.50%), and 72 h (82.36%) after the Al treatment was applied. Compared to the control, the length of the primary root was significantly shorter 4, 8, 24, 48, and 72 h after Al treatment (49.81%, 66.46%, 66.51%, 73.00%, and 76.80%, respectively).

The activities of both antioxidant enzymes and the concentrations of ROS and MDA were higher under Al treatment. Al stress also significantly induced the activities of SOD and POD (26.32% and 10.00%, respectively). In addition, the amount of ROS also was significantly elevated by 16.10%, while the amount of MDA did not change significantly (8%) when plants were grown in a simplified nutrient solution containing 20 µM AlCl_3_ (Table 4).

The correlations between the measured parameters under Al treatment for 25 bean cultivars showed that ROS positively correlated with SOD (*p* ≤ 0.01), POD (*p* ≤ 0.05), and MDA (*p* ≤ 0.01). Additionally, positive correlations were observed between ROS and root length 4, 8, and 24 h after the Al treatment. A highly significant correlation (*p* ≤ 0.001) was observed between SOD and POD (positive). MDA positively correlated with root DW and root volume change 72 h after Al treatment (Table 5).

## 3. Discussion

Anthropogenic pollution is becoming more relevant than ever in the 21st century because of the constantly growing industrial production to satisfy the demand for food, energy, and living conditions of the continuously growing human population [70,71]. Aluminum toxicity is one of the side effects of soil acidification, where the soil pH is less than or reaches 5.5, which can cause growth inhibition, i.e., reduced growth in the roots, stems, and shoots, decreased biomass production, and serious yield loss [72]. This phenomenon occurs particularly in the subtropical and tropical regions, but soil acidification is also observed in European soils [72].

According to several research studies, the root is the most sensitive plant organ to Al toxicity [7,20,65,67,69,72] because of the direct contact it has with Al in the soil. Results of this experiment confirm such observations as lower root dry biomass and root-to-shoot ratio were observed in 23 of 25 common bean cultivars (except AC Island and Arapaho) under Al treatment (Table 1). The reduced root biomass could be associated with damage to the root cell wall [73] and plasma membrane [74], a decrease in the amount of hemicellulose and pectin fractions in the roots [75], which makes them rigid [76], calmodulin changes in the symplast [77], or imbalanced nutrient uptake [78]. Such factors have a significant impact on plant growth. Selective inhibition of the root and shoot dry weight of the common bean cultivars further show that the response of common beans to Al stress is genotype-specific. Considering the stability of root-to-shoot dry mass of Arapaho and AC Island under Al treatment, these cultivars could potentially be used in the future production of common beans under Al toxic soils and be included in Al tolerance breeding programs. In addition, the change in root volume percentage was very low for Arapaho at 24, 48, and 72 h after Al treatment in contrast to other cultivars (Table 2), which further suggests this cultivar is a better alternative for cultivation under Al toxicity.

Growth requires two main constituents: cell division and cell elongation. A short-term exposure (30–90 min) to Al treatment inhibited root elongation in common beans [79]. Aluminum-inhibited root elongation is an appropriate parameter for the classification of the investigated genotypes related to Al sensitivity or Al resistance [80]. In a set of 28 Andean and Mesoamerican common bean genotypes, nine genotypes were Al-sensitive (Al-inhibited root elongation >50%), 12 genotypes had intermediate sensitivity (Al-inhibited root elongation 30–50%), and seven genotypes were Al-resistant (Al-inhibited root elongation ≤30%) [80]. In this study, six cultivars were Al-sensitive and indicated ≥90% Al-induced primary root elongation inhibition 72 h after Al treatment (Aztec was among these cultivars). Additionally, Al-induced root elongation inhibition was between 70% and 90% in 14 cultivars, two cultivars had 50–70% inhibition, and three were intermediate Al-sensitive (30–50%). In agreement with the results on root and shoot dry weight, as well as on the percentage change in root volume above, Arapaho had the least root inhibition (8–72 h after Al treatment) compared to the 24 other cultivars (Table 3). This further confirms that this cultivar is less sensitive to Al stress, which could result in better growth.

Zheng and Yang [81] stated that Al toxicity modified cell-wall characteristics and caused oxidative stress. Aluminum toxicity leads to oxidative stress; its degree depends on the concentration of Al and the sensitivity of plants. When antioxidant systems are insufficient, more reactive oxygen species (ROS) are generated, leading to oxidative stress in plants [82]. Nahar et al. [83] studied the effect of Al on ROS production in mung beans. They found that Al toxicity caused higher ROS production, resulting in higher lipid peroxidation in membranes. In addition, elevated ROS quantities were found in pea roots, and the ROS production increased with the duration of exposure. Yamamoto et al. [84] suggested that increased amounts of ROS inhibit the root elongation of peas. In this study, ROS production under Al stress varied with cultivars, whereby nine cultivars had significantly higher accumulation (23.62–72.99% increase). Noticeably lower ROS concentrations were measured in Flint and Montrose (27.81% and 46.55%, respectively) under Al treatment in this study. Interestingly, Arapaho was among the cultivars with low ROS accumulation under Al treatment (Figure 1). This corresponds well with the observed decrease in the inhibition of the root parameters, implying that this cultivar had a better mechanism of avoiding excessive production of ROS. Although excessive ROS accumulation could be detrimental to plants, when produced in lower quantities, they (especially hydrogen peroxide) could act as signaling molecules to switch on the plant’s defense responses during abiotic stress conditions [85]. This could be the case for Arapaho because of the significant correlation between ROS and root length between 4 and 24 h post Al treatment (Table 5).

Antioxidant enzymes are very important in regulating the ROS levels in plants. Zhang et al. [86] found that the activity of SOD was significantly higher in fava bean roots 6 days after 50 and 100 µM Al treatments, and 9 days after 100 µM Al treatment relative to non-Al-treated control. Additionally, the activity of POD also increased after 100 µM Al treatment compared to the control. An Al-tolerant broad bean cultivar had significantly higher SOD activity 12 and 24 h after 50 µM Al treatment, while the activity decreased in an Al-sensitive cultivar. The activity of POD was higher in both cultivars 2, 4, 8, and 12 h after Al treatment, and it was notably higher in the Al-sensitive cultivar than the Al-tolerant one [87]. However, in maize, the activities of both SOD and POD were noticeably higher in Al-sensitive maize and did not change in the Al-resistant cultivar after Al treatment [45]. This further confirms the importance of a cultivar type and its sensitivity to Al. In the current study, SOD activity was higher in all cultivars under Al stress conditions. Twenty of the 25 cultivars had significantly higher activity, varying between 17.86% and 45.50% increases. Although SOD activity was significantly increased by Al treatment in Arapaho, the level was not high (Figure 2). This suggests that the lower level of ROS observed above for this cultivar could be maintained by other antioxidant systems. However, this needs to be elucidated further. Another explanation could be that the low ROS level was not caused by higher antioxidant activity but by lower Al uptake due to better Al chelation in the rhizosphere and apoplast by organic acid exudations [88]. Although there was a positive correlation (*p* ≤ 0.01) between SOD and the ROS, it is unlikely that SOD activity was directly involved in the defense against Al toxicity because it did not correlate well with any of the root and shoot parameters (Table 5).

A differential response for POD activity under Al treatment was observed in common bean cultivars. Some cultivars had significantly high POD activity (12 cultivars) while others had lower activity under Al treatment. The role of POD in improving the tolerance of common bean cultivars to Al stress is uncertain because Arapaho (which was found to be less Al-sensitive) was among the cultivars with reduced POD activity. Moreover, a cultivar with the highest POD activity (cultivar Windbreaker) (Figure 3) had the highest recorded ROS accumulation, showing that POD did not effectively reduce ROS accumulation. Furthermore, a significantly positive correlation between POD activity (Table 5) and ROS shows that its activity increased with an increase in ROS accumulation under Al stress. Therefore, there is no strong evidence that POD has a concrete role in improving the performance of common bean plants during Al stress.

The measurement of lipid peroxidation is one of the indices of stressed conditions. A higher rate of lipid peroxidation was observed in many plants under Al stress, e.g., pea roots [89], soybean root tips [90], and fava bean roots [86,87]. The generated amount of MDA during lipid peroxidation increased with the applied Al concentration [91]. In contrast, Yamamoto et al. [89] observed higher MDA concentration only in the root apex of peas under Al toxicity, and they stated that the elevated rate of lipid peroxidation and the higher MDA levels are not the direct cause of Al toxicity. Zhang et al. [86] investigated the impacts of Al toxicity on fava bean in a 9 day hydroponic experiment. They used 10, 50, and 100 µM Al concentrations and found that MDA content was significantly higher in the leaves and roots after 6 and 9 days of 50 and 100 µM Al treatment. Additionally, the amount of MDA in broad bean roots also increased 2, 4, 8, 12, and 24 h after 50 µM Al application. The increase was higher in the case of the Al-sensitive cultivar compared to the Al-tolerant one [87]. Chen et al. [87] concluded that oxidative stress was generated by Al toxicity, through elevated lipid peroxidation. As with many abiotic stresses, MDA levels increased with Al stress for most cultivars (Figure 4). Arapaho had the highest reduction in MDA concentration under Al treatment, as well as the highest MDA concentration under nontoxic (control) conditions. This suggests that Arapaho is sensitive to acidic pH growing conditions and that Al alleviated its proton toxicity stress [92]. Furthermore, a positive significant correlation was observed between MDA and ROS, showing that Al stress induced the accumulation of ROS along with an increase in lipid peroxidation. Although POD had no clear role in lessening the sensitivity of common beans to Al toxicity, it somehow contributed to reducing lipid peroxidation (Table 5). Although lipid peroxidation is crucial for the cellular membranes, it does not always have a direct impact on root growth and elongation [91]. A highly significant positive correlation was observed between POD and SOD (*p* ≤ 0.001), and significant positive correlations were observed between ROS and SOD, and between SOD and MDA (*p* ≤ 0.01) In addition, MDA and POD were significantly (*p* ≤ 0.01) negatively correlated in common beans under Al treatment, which is in agreement with such findings.

## 4. Materials and Methods

### 4.1. Growth Conditions

The surface of 25 common bean cultivar seeds (from US commercial lines that were developed by US bean breeders) were scratched to abrade the seed coat and were germinated for 5 days between two wet sheets of filter paper oriented vertically. Seedlings were transferred to a simplified nutrient solution with compositions of 0.5 mM CaCl_2_, 0.5 mM KCl, and 8 μM H_3_BO_3_ [80] in black plastic pots holding 4.5 L of solution and equipped with an aerator. Seedling roots were placed in the nutrient solution, and shoots were maintained above the solution using one-holed cups placed in the lids on top of the pots. The chemical composition of the hydroponic solution ensured optimal root growth for a minimum of 3 days. The pH of the nutrient solution was lowered with 0.1 M HCl to pH 5.0 after 24 h. The pH was adjusted to 4.5 after another 24 h and monitored daily, while being maintained at 4.5 using 0.1 M HCl or 0.1 M KOH, as needed, throughout the study. There were two treatments (0 and 20 μM AlCl_3_), each consisting of three pots that contained four plants. Seven plants were used for morphological and weight measurements, and five were used for enzymes assays. The pots were placed in a grid-like fashion while making sure that there was no set order to them with respect to the cultivar or the treatment in order to create a completely random design for the experiment. Common beans were cultivated in a growth chamber with the following environmental conditions: daytime lighting 16 h, dark period 8 h, day temperature 20 °C, night temperature 15 °C, relative humidity 50% ± 5%, and photon flux density at the top of plants of 574 μmol·m^−2^·s^−1^.

### 4.2. Root Elongation

In order to prevent damage, the primary root of each plant was marked 2 cm from the tip of the root with a permanent marker 2 h before the Al treatment. Subsequently, beans were placed into a nutrient solution that either contained 20 μM AlCl_3_ or none. Measurements (in mm) of root elongation of the primary root were taken 4, 8, 24, 48, and 72 h after Al treatment and rounded to the nearest mm. Measurements were made from the permanent marker to the root tip.

Change in root volume was estimated using the Archimedes method on a day-to-day basis. This is a quick and precise technique to measure root growth without any root damage [92].

### 4.3. Dry Weight 

The dry weight of each sample was determined using the thermogravimetry method. The fresh roots and shoots of 25 common beans per plant were oven-dried for 72 h at 60 °C. After the samples cooled to room temperature, they were weighed using an analytical scale. The root/shoot ratios were calculated for each cultivar and both treatments. The ratios are based on the dry weight of the roots to the dry weight of the shoots.

### 4.4. Enzymes Assays

The following procedure was used for the preparation of the enzyme extract: liquid nitrogen was used to grind frozen bean root tissue (400 mg) to a fine powder and homogenized in 50 mM phosphate buffer (pH 7.8, 2 mL) containing 0.1 mM ethylenediaminetetraacetic acid (EDTA), 1% polyvinylpyrrolidone (PVP) (*w*/*v*), and 1 mM phenylmethylsulfonyl fluoride (PMSF). The extract was centrifuged at 10,000× *g* for 15 min, and the supernatant was used as the enzyme extract for superoxide dismutase (SOD), guaiacol peroxidase (POD), and reactive oxygen species (ROS).

The activity of SOD was measured according to the methods of Giannopolities and Ries [93] and Beyer and Fridovich [94]. One SOD unit was determined as the amount of enzyme needed to inhibit 50% of nitroblue tetrazolium (NBT) light-induced reduction compared to the test tubes that did not contain the plant extract. After centrifugation, 25 µL off supernatant as plant extract, 25 µL of NBT (9 mM), 25 µL of riboflavin (0.25 mM), 250 µL of methionin (0.16 M), and 2.675 mL of phosphate buffer (pH 7.8, 50 mM) were mixed and kept at room temperature; then, the mixture’s absorbance was measured after 15 min at 560 nm. The blank tubes contained 2.7 mL of phosphate buffer and no plant extract; all other components were the same as described above.

The POD assay as proposed by Zieslin and Ben-Zaken [95] was adopted for evaluating the activity of guaiacol peroxidase. The modified mixture had 50 μL of 0.2 M H_2_O_2_, 100 μL of 50 mM guaiacol, 340 μL of distilled H_2_O, 500 μL of 80 mM phosphate buffer (pH 5.5), and 10 μL of enzyme. The POD activity was determined on the basis of the concentration of generated tetraguaiacol. The absorbance of the reaction mixture was read at 470 nm for 3 min at 30 °C. All the above mentioned chemicals were used for the blank, but 50 mM phosphate buffer was used instead of the enzyme. The concentration of tetraguaiacol was determined using a 25.5 mM^−1^·cm^−1^ extinction coefficient.

The method used for the determination of the total amount of ROS (reactive oxygen species) was adopted from Keller et al. [96]. This method is based on the oxidation of the nonfluorescent 2.7-dichlorodihydrofluorescein to the highly fluorescent 2.7-dichlorofluorescein by ROS. Ten microliters of 2.7-dichlorofluorescein diacetate (10 µM) and 100 µL of plant extract were transferred to a 96-well flat-bottom microplate and incubated for 1 h at room temperature; then, fluorescence intensity was measured using a Synergy HT microplate reader (Biotek Instrument, Inc., Winooski, VT, USA) with 485 nm as the excitation and 530 nm as the emission wavelength.

To determine the amount of malondialdehyde (MDA) in bean roots, the thiobarbituric acid (TBA) test was used according to Heath and Packer [97]. For measurement, 100 mg of frozen bean roots were crushed with liquid nitrogen and homogenized to a fine pulp on ice in 1 mL of 0.25% TBA and 10% trichloroacetic acid (TCA), followed by centrifugation (10,800× *g* for 25 min at 4 °C). Thereafter, the supernatant (0.2 mL) was transferred to clean Eppendorf tubes. To this, a 0.8 mL mixture of 20% TCA and 0.5% TBA was added to the tubes. The mixtures were vortexed and warmed at 95 °C for 30 min and cooled on ice. The tubes were centrifuged again at 10,800× *g* for 10 min at 4 °C, and the absorbances were measured at 532 nm and 600 nm. An extinction coefficient of 155 mM^−1^·cm^−1^ was used to quantify MDA.

### 4.5. Data Evaluation 

Research data were analyzed for normal distribution using the Kolmogorov–Smirnov and Shapiro–Wilk tests [98]. The Mann–Whitney U nonparametric test (*p* < 0.05) was used to compare the mean values [99]. Significant differences were denoted by lowercase letters. Relationships between two measured parameters were computed by Pearson correlation [100]. The statistical analysis was performed using IBM SPSS Statistics 25 software (Armonk, NY, USA). 

## 5. Conclusions

To conclude, 20 µM AlCl_3_ treatment had significant negative effects on most of the measured characteristics, except shoot dry weight and MDA concentration, as well as biochemical and physiological parameters of the different cultivars’ roots. The average values in Table 4 of Al-treated plants show that the activities of SOD and POD, and the total number of ROS values were significantly higher, while the root dry biomass, root-to-shoot ratio, the growth of primary root length, and root volume were significantly lower. According to the results of this study, Arapaho and AC Island cultivars could potentially be used in the future production of common beans in aluminum-toxic soils. Therefore, these two cultivars could also be included in Al tolerance breeding programs.

## Figures and Tables

**Table 1:** The effect of AlCl_3_ on the dry weight of roots and shoots (DW) (mg·plant^−1^), as well as the root-to-shoot ratio, of 25 common bean cultivars grown for 3 days in hydroponics.

Cultivars	Root DW (mg·plant^−1^)		Shoot DW (mg·plant^−1^)		Root/Shoot Ratio	
	0 µM Al	20 µM Al	0 µM Al	20 µM Al	0 µM Al	20 µM Al
AC Island	76 ± 18	69 ± 11	273 ± 52	282 ± 28	0.28	0.24
Apache	77 ± 2	41 ± 4 *	269 ± 33	267 ± 52	0.28	0.15 *
Arapaho	71 ± 27	52 ± 11 *	227 ± 44	238 ± 48	0.26	0.22
Aztec	71 ± 7	31 ± 1 *	261 ± 20	227 ± 38 *	0.27	0.14 *
Bill Z	85 ± 22	53 ± 5 *	224 ± 46	240 ± 40	0.38	0.22 *
Buckskin	80 ± 24	45 ± 6 *	271 ± 70	254 ± 18	0.29	0.18 *
Burke	64 ± 4	31 ± 5 *	263 ± 21	272 ± 37	0.24	0.11 *
Croissant	104 ± 15	46 ± 1 *	268 ± 36	250 ± 24	0.39	0.18 *
Flint	58 ± 13	33 ± 6 *	208 ± 47	237 ± 44	0.28	0.14 *
Fargo	90 ± 15	57 ± 7 *	271 ± 59	303 ± 26	0.33	0.19 *
Grand Mesa	77 ± 11	49 ± 3 *	241 ± 24	246 ± 8	0.32	0.09 *
Kimberly	60 ± 15	26 ± 6 *	307 ± 45	288 ± 19	0.19	0.09 *
Kodiak	93 ± 14	53 ± 14 *	305 ± 34	264 ± 40	0.30	0.20 *
Max	103 ± 12	59 ± 12 *	271 ± 28	270 ± 5	0.38	0.22 *
Montrose	59 ± 10	32 ± 6 *	250 ± 19	28 ± 6 *	0.24	0.11 *
La Paz	75 ± 11	45 ± 6 *	206 ± 19	229 ± 37	0.36	0.20 *
Ouray	103 ± 19	78 ± 15 *	237 ± 45	251 ± 25	0.43	0.31 *
Poncho	78 ± 20	48 ± 7 *	304 ± 48	326 ± 52	0.26	0.15 *
Pinto	65 ± 10	35 ± 2 *	247 ± 30	268 ± 26	0.26	0.13 *
Quincy	67 ± 5	26 ± 4 *	276 ± 25	271 ± 37	0.24	0.10 *
Santa Fe	93 ± 11	53 ± 1 *	308 ± 36	279 ± 17	0.30	0.19 *
Sierra	89 ± 13	50 ± 6 *	240 ± 21	236 ± 19	0.37	0.21 *
TARS-09	68 ± 7	30 ± 8 *	189 ± 34	221 ± 38	0.36	0.14 *
Topaz	78 ± 4	46 ± 1 *	234 ± 26	247 ± 35	0.33	0.19 *
Windbreaker	72 ± 7	46 ± 1	258 ± 29	264 ± 2	0.28	0.18 *

Values in columns are means ± standard deviation (*n* = 7); DW: dry weight. * Significant differences compared to control based on Shapiro–Wilk test (*p* ≤ 0.05).

**Table 2:** The change in root volume percentages of 25 Pinto bean cultivars 24, 48, and 72 h after AlCl_3_ treatment, relative to non-stressed controls.

Cultivars	% Δ of Root Volume		
	24 h after Al Treatment	48 h after Al Treatment	72 h after Al Treatment
AC Island	−41	−53	−65
Apache	−68	−72	−83
Arapaho	+11	−39	−53
Aztec	−49	−62	−77
Bill Z	−47	−53	−66
Buckskin	−70	−58	−69
Burke	−82	−84	−89
Croissant	−81	−80	−83
Fargo	−54	−67	−69
Flint	−55	−70	−69
Grand Mesa	−35	−53	−59
Kimberly	−73	−90	−92
Kodiak	−46	−60	−71
La Paz	−61	−72	−77
Max	−69	−74	−76
Montrose	−56	−73	−80
Ouray	−46	−59	−63
Poncho	−63	−73	−81
Pinto	−65	−82	−87
Quincy	−26	−79	−86
Santa Fe	−31	−60	−76
Sierra	−36	−63	−68
TARS-09	−69	−85	−85
Topaz	−37	−69	−66
Windbreaker	−55	−68	−73

**Table 3:** The root length inhibition percentage of 25 common bean cultivars 4, 8, 24, 48, and 72 h after AlCl_3_ treatment, relative to non-stressed controls.

Cultivars	Al-Induced Root Length Inhibition as a Percentage (%)				
	0–4 h	4–8 h	8–24 h	24–48 h	48–72 h
AC Island	30	58	19	31	56
Apache	55	74	81	89	91
Arapaho	49	48	−6	8	35
Aztec	62	46	82	95	97
Bill Z	30	36	7	22	51
Buckskin	72	79	84	85	77
Burke	15	76	78	87	96
Croissant	46	77	68	86	75
Fargo	40	80	83	74	81
Flint	65	69	86	81	82
Grand Mesa	54	46	26	31	39
Kimberly	67	85	90	93	96
Kodiak	39	78	68	78	71
La Paz	37	71	73	75	79
Max	57	68	77	84	82
Montrose	53	77	80	92	88
Ouray	24	55	21	19	48
Poncho	39	79	9	94	92
Pinto	42	57	85	85	76
Quincy	51	7	76	75	78
Santa Fe	56	83	76	79	74
Sierra	62	84	80	90	87
TARS-09	56	77	80	42	89.
Topaz	35	86	77	94	90
Windbreaker	49	85	82	78	86

**Table 4:** Average values of measured parameters of 25 Pinto cultivars based on the applied treatments (0 µM AlCl_3_ and 20 µM AlCl_3_): root and shoot dry weight *n* = 175 ± SD; root volume and root length *n* = 300 ± SD; SOD, POD, ROS, and MDA, *n* = 125 ± SD.

	Treatment	
	0 µM AlCl_3_	20 µM AlCl_3_
Root DW	0.08 ± 0.02	0.05 ± 0.02 *
Shoot DW	0.26 ± 0.05	0.26 ± 0.04 ns
Root:shoot	0.31 ± 0.07	0.18 ± 0.06 *
Δ root volume cm^3^/24 h	0.29 ± 0.15	0.14 ± 0.11 *
Δ root volume cm^3^/48 h	0.40 ± 0.16	0.11 ± 0.09 *
Δ root volume cm^3^/72 h	0.34 ± 0.17	0.06 ± 0.05 *
PRG 4 h after Al (mm/h)	5.14 ± 2.14	2.58 ± 1.44 *
PRG 8 h after Al (mm/h)	7.99 ± 3.25	2.68 ± 1.51 *
PRG 24 h after Al (mm/h)	27.68 ± 3.96	9.27 ± 8.99 *
PRG 48 h after Al (mm/h)	42.64 ± 6.97	11.53 ± 11.61 *
PRG 72 h after Al (mm/h)	41.20 ± 6.78	9.56 ± 8.66 *
SOD	0.14 ± 0.03	0.19 ± 0.04 *
POD	4.41 ± 2.89	4.88 ± 2.61 *
ROS	712,864.92 ± 407,069.50	849,014.63 ± 352,566.80 *
MDA	13.97 ± 9.61	15.12 ± 10.05 ns

DW: dry weight, PRG: primary root growth, SOD: superoxide dismutase, POD: peroxidase, ROS: reactive oxygen species, MDA: malondialdehyde. * Significant differences compared to control based on Shapiro–Wilk test (*p* ≤ 0.05); ns: not significant.

**Table 5:** Correlations based on the average values for all the cultivars combined under AlCl_3_ treatment.

Char I	Characteristics II													
	Root DW	Root Shoot	Root Volume 24 h	Root Volume 48 h	Root Volume 72 h	Root Length 4 h	Root Length 8 h	Root Length 24 h	Root Length 48 h	Root Length 72 h	ROS	SOD	POD	MDA
Root DW	1	ns	ns	ns	ns	ns	ns	ns	ns	ns	ns	ns	ns	+0.151 *
Root:Shoot		1	ns	ns	ns	ns	ns	ns	ns	ns	ns	ns	ns	ns
Root volume 24 h			1	ns	ns	ns	ns	ns	ns	ns	ns	ns	ns	ns
Root volume 48 h				1	+0.233 *	ns	ns	ns	ns	ns	ns	ns	ns	ns
Root volume 72 h					1	ns	ns	ns	ns	ns	ns	ns	ns	+0.169 *
Root length 4 h						1	+0.231 ***	+0.206 **	ns	−0.345 ***	+0.165 *	ns	ns	ns
Root length 8 h							1	+0.173 *	+0.135 *	ns	+0.121 *	ns	ns	ns
Root length 24 h								1	ns	ns	+0.179 *	ns	ns	ns
Root length 48 h									1	ns	ns	ns	ns	ns
Root length 72 h										1	ns	ns	+0.126 *	ns
ROS											1	+0.195 **	+0.179 *	+0.202 **
SOD												1	+0.256 ***	ns
POD													1	−0.430 **
MDA														1

DW: dry weight, SOD: superoxide dismutase, POD: peroxidase, ROS: reactive oxygen species, MDA: malondialdehyde, Char: characteristics. * *p* ≤ 0.001, ** *p* ≤ 0.01, *** *p* ≤ 0.05; ns: not significant.

## Data Availability

The data presented in this study are available upon request from the corresponding author. All data, tables and figures presented in this manuscript are original.
